# Vital Recorder—a free research tool for automatic recording of high-resolution time-synchronised physiological data from multiple anaesthesia devices

**DOI:** 10.1038/s41598-018-20062-4

**Published:** 2018-01-24

**Authors:** Hyung-Chul Lee, Chul-Woo Jung

**Affiliations:** Department of Anesthesiology and Pain Medicine, Seoul National University College of Medicine, Seoul National University Hospital, Seoul, Republic of Korea

## Abstract

The current anaesthesia information management system (AIMS) has limited capability for the acquisition of high-quality vital signs data. We have developed a Vital Recorder program to overcome the disadvantages of AIMS and to support research. Physiological data of surgical patients were collected from 10 operating rooms using the Vital Recorder. The basic equipment used were a patient monitor, the anaesthesia machine, and the bispectral index (BIS) monitor. Infusion pumps, cardiac output monitors, regional oximeter, and rapid infusion device were added as required. The automatic recording option was used exclusively and the status of recording was frequently checked through web monitoring. Automatic recording was successful in 98.5% (4,272/4,335) cases during eight months of operation. The total recorded time was 13,489 h (3.2 ± 1.9 h/case). The Vital Recorder’s automatic recording and remote monitoring capabilities enabled us to record physiological big data with minimal effort. The Vital Recorder also provided time-synchronised data captured from a variety of devices to facilitate an integrated analysis of vital signs data. The free distribution of the Vital Recorder is expected to improve data access for researchers attempting physiological data studies and to eliminate inequalities in research opportunities due to differences in data collection capabilities.

## Introduction

An increasing number of hospitals have adopted commercial anaesthesia information management system (AIMS) for clinical use^1^. The use of commercial AIMS has significantly improved the cost reduction, operations management, quality of care, safety, documentation, and research^2^. The automated anaesthesia record, a basic component of modern AIMS, has greatly contributed to the clinical decision support and patient outcomes research, by providing large amounts of complete, comprehensible, and objective physiological data^3–5^. However, AIMS demonstrates limited capability for the acquisition of vital signs data, owing to its limitations with regard to the time resolution, number of parameters recorded, inability to record data from multiple equipment, and high cost.

Based on the hypothesis of a high demand for tools that enable researchers to easily record and manipulate high-quality vital signs data for prospective and retrospective studies, we developed the Vital Recorder, a free Windows program that automatically collects high-resolution time-synchronised vital signs data generated from various anaesthesia equipment for research purpose. This program can provide high-quality data for researchers at tertiary hospitals, and research opportunities for individual researchers who are not using AIMS. In the current study, a detailed description of the characteristics of the Vital Recorder and our experience from over eight months of operation in the clinical practice are reported.

## Results

Data collection from surgical patients was performed in five operating rooms from June 2016 to July 2016, and in 10 operating rooms from August 2016 to January 2017. For the 4,335 surgical cases, the automatic recording generated 7,404 files, and 4,272 (98.5%) cases were successfully retrieved. Of the 63 recording failures, 44 were due to automatic Windows^®^ updates and 19 were due to the unintentional disconnection of the data cable. Partial loss of data was observed in 147 out of the 4,272 (3.4%) cases.

Out of the automatically recorded 4,272 cases, 3,701 cases were elective operations and 571 cases were emergency operations. There were 4,073 general anaesthesia cases and 199 regional anaesthesia cases. The retrospective analysis of data tracks showed that general anaesthesia was performed with volatile anaesthesia (2,078 cases, 51.0%) or total intravenous anaesthesia (1,995 cases, 49.0%). The total recorded time was 13,489 h, and the time per case was 3.2 ± 1.9 h. The list of medical devices, number of cases, and types of collected data are described in detail (Table 1).

**Table 1:** Results of automatic data recording over eight months.

Device	Collected data	Number of cases
Solar 8000	numeric data (every 2 sec)	4272
TramRac 4 A	ECG and plethysmography waves (500 Hz)	4272
	Arterial pressure wave (500 Hz)	2348
	Central venous pressure wave (500 Hz)	1126
Primus	Capnography wave (62.5 Hz)numeric data (every 7 sec)	1150
BIS Vista^TM^	2-channel EEG wave (128 Hz)numeric data (every 1 sec)	1093
Orchestra^®^	numeric data (every 1 sec)	674
Vigileo, EV 1000, Vigilance II	numeric data (every 2 sec)	151
CardioQ-ODM+	pressure and flow waves (180 Hz)numeric data (every 1 sec)	8
INVOS^TM^	numeric data (every 5 sec)	1
Belmont^®^ Rapid Infuser	numeric data (every 2.875 mL)	3

## Discussion

The authors developed a Windows program for the automatic recording of high-resolution time-synchronised vital signs data from various anaesthesia equipment, and successfully performed automated recording in 98.5% of the cases during eight months of operation.

A recent review of the perioperative patient clinical outcomes study using AIMS-based data identified 21 studies conducted in academic university hospitals from 1980 to 2013^5^. In this paper, the authors pointed out that while AIMS provided accurate records, especially highly reliable physiological data from the patient monitoring system, there was a risk that the unprocessed artefacts recorded by AIMS might affect study results. These artefacts included heart rate and ST segment errors due to electrocautery interference and patient’s movement, oxygen saturation measurement errors due to dislocation of sensor or vasopressor use, non-invasive blood pressure errors caused by leaning on the pressure cuff or misplacement of the cuff, and invasive arterial pressure misdiagnosed by flushing and blood sampling^6^. Such falsely detected artefacts have been a major drawback of computerised recording^7^. A visual check of the recorded data is essential to eliminate these artefacts; however, this can be time-consuming for large datasets^8^. The most commonly used method of artefact removal is using a median or mean value for a certain time range^9^. Kool and colleagues^10^ reported that using a median of 1 minute epoch for patient monitoring data acquired every 5 s provided reliable data for heart rate and oxygen saturation, and acceptable reliability for non-invasive blood pressure values. In general, oversampling reduces aliasing problems and improves the signal-to-noise ratio. However, since the current AIMS is designed solely for medical records, measurements are commonly recorded at very low sampling frequencies such as every 1–5 min. Using a median filter to eliminate artefacts inevitably leads to an excessive reduction in the amount of data, resulting in new artefacts due to unintended removal of key data. Current low-resolution AIMS data are not suitable for studies that observe changes within 5 min, for example, those measuring the effect of vasopressors on postreperfusion syndrome in liver transplantation^11^.

There have been several programs that can complement the shortcomings of AIMS. A specific program can be considered to acquire accurate vital signs data from the patient monitor. For example, Datex-Ohmeda S/5™ Collect program (GE Healthcare, Waukesha, WI, USA) can obtain high-resolution data from the Datex-Ohmeda S/5™ series monitors. However, the program was built for Windows XP and does not work on the current Windows operating systems, and the manufacturer does not intend to update it. The latest commercial program ixTrend (ixellence GmbH, Wildau, Germany) can be used to obtain data from Philips monitors only. However, the use of these commercial programs is costly for individual researchers or for big data collection from multiple monitors. As alternatives, a few programs were created and used by researchers. Kool and colleagues^10^ reported that they collected numerical data at five-second intervals from the Datex Ohmeda S/5™ monitoring system using their own AIMS (Vierkleurenpen^©^, version 1.4.5, 2010). Liu and colleagues^12^ also reported the collection of vital signs data of 32 surgical patients, from Philips IntelliVue MP series monitors, using a self-developed program. However, these authors did not disclose their programs. Karippacheril and Ho^13^ reported VSCapture, a simple open-source tool that enabled data collection from the Datex Ohmeda S/5™ monitor, and released the source code. On the other hand, the data from equipment other than the patient monitors could be obtained with high time resolutions, by using proprietary programs provided by the manufacturers, such as TrendCom (Masimo corporation, Irvine, CA, USA) and MultiDataLogger (Edwards Lifesciences corporation, Irvine, CA, USA). If manufacturers do not provide a program for real-time data acquisition from the device such as the BIS monitor, a program such as the BSA A2000 developed by a private researcher could be used (http://www.med.osaka-u.ac.jp/pub/anes/www/software/software.E.html, accessed 4 January 2012). However, it has been pointed out that data obtained through these independent programs have problems of time synchronisation with the patient monitoring data^14^. A commercial program such as Rugloop II (Demed Bvba, Temse, Belgium) or a public program such as the Monitor program (https://www.cuhk.edu.hk/med/ans/softwares.htm, accessed 1 January 2016) can be used to acquire synchronised data from various devices. While these programs are specialised for the acquisition of synchronised data, they appear to lack data editing capabilities and add-ons for medical researchers.

As described in the Methods section, the Vital Recorder provides superior functionality and extensibility over the existing free or commercial programs, by including time-synchronised capture of data from various devices, filters for data analysis, and remote monitoring functions. In addition, a special advantage of the Vital Recorder is its user interface and automated recording. Many medical researchers who would like to use vital signs data are often unfamiliar with data manipulation. Public programs are frequently user-unfriendly, and expensive commercial programs have complex functions that are difficult to learn. Therefore, we aimed to improve usability by introducing an interface similar to that of a video-editing program. As data can be manipulated as easily as editing video clips, it is expected that anyone can quickly and easily use high-resolution physiological data having more than 100 data tracks. Furthermore, the Vital Recorder’s automatic recording capability is superior to that of commercial AIMS in terms of high-resolution data acquisition, and is stable enough to collect more than 4000 cases with a success rate of 98.5% in daily operations. We assume that the Vital Recorder is currently the most powerful data collection tool for use in prospective or retrospective studies using vital signs big data because of the ease of data collection and high fidelity of the recorded data.

However, there are some limitations for the use of the Vital Recorder. The first is that some devices are not yet supported, owing to unidentified communication protocols. We are constantly updating the protocols to support more anaesthesia devices in the future. The second is data fragmentation. In addition to the breaks between patient cases during automatic recording, data gaps may occur within the data, resulting in fragmented files^15^. The main reason for this appears to be the unblocked electrical noise, because the data gap occurred frequently during excessive use of electrocautery, especially in old operating rooms where electromagnetic shielding was not working perfectly^16^. Fragmentation was reduced when shielded filtering cables or a power source separate from the electrocautery source was used. We also provide a data-merge function to combine fragmented files into a single case. The third problem is excessive data volume. Cost-related issues are less important because storage media are becoming less expensive. However, while the Vital Recorder has the advantage of recording all parameters, it also has the disadvantage of handling all parameters. Even when only a part of the data tracks is needed, a large file should be opened and all parameters must be loaded. This problem can be solved by using our utility programs that provide information about the parameters inside the file and extract the queried data tracks without opening the file. Finally, the Vital Recorder does not add site identifiers to recording files to protect the privacy of personal health information because the program is deliberately designed for non-clinical research purposes. In conducting studies, the researchers need to manage individual patient-specific information that can be matched to the recorded vital files.

In conclusion, the Vital Recorder’s automatic recording and remote monitoring functions help to build physiological big data with minimal effort and low cost. The free distribution of the Vital Recorder is expected to contribute to eliminating inequalities in research opportunities due to data acquisition capabilities, and enhancing data accessibility for small and medium hospitals and individual researchers planning biomedical research.

## Methods

### Approval for data collection using the Vital Recorder

The Institutional Review Board of the Seoul National University Hospital approved a prospective data registry study entitled ‘Registry Construction of Intraoperative Vital Signs and Clinical Information for Retrospective Cohort Study in Surgical Patients’ (H-1408-101-605). Data collection was performed in accordance with relevant guidelines and regulations of the committee. The study plans to collect vital signs data from surgical patients over the next 10 years. The study was also registered at clinicaltrials.gov (NCT02914444). Written informed consent was waived due to anonymity of the data.

### Description of Vital Recorder program

#### Overview

The Vital Recorder is a stand-alone program written in C++, which runs on the Windows operating system version Vista or later. The Vital Recorder is very light, with an installed size of less than 10 MB and CPU usage less than 5% on dual core Pentium systems during recording. The recording and editing of vital signs data can be performed within the Vital Recorder program, and four additional utility programs assist in data management. A graphical user interface similar to that of a video-editing program has been adopted so that researchers who are not familiar with data-management programs can easily manipulate the vital signs data. The recorded data can be exported to various machine or human readable file formats. The Vital Recorder has options to automatically record each patient case separately and to remotely monitor the status of data collection. The program, sample files, and an overview video are accessible from the publicly open data repository (https://osf.io/uq2p2) or from the authors’ own website (https://vitaldb.net).

#### Hardware requirements

The minimum-required hardware for data recording is a computer with an Intel^®^ Atom^®^ processor, 512 MB RAM, and 100 MB free hard-disk space; however, it is recommended to expand the hardware for seamless data editing.

#### Supported anaesthesia equipment

While there is no limit to the number of devices that can be connected to the Vital Recorder at the same time, two to six devices are generally used simultaneously during routine patient care. Currently, more than 20 anaesthetic devices from 10 major companies are supported, and the number of devices on the list is increasing constantly (Table 2). Communication between the various medical devices and the computer is established via an RS-232C serial connection. Analog-to-digital converters may be required to obtain waveform signals from the analog port of the patient monitor (Tram module of Solar^TM^ 8000 patient monitor, GE healthcare, Wauwatosa, WI, USA). After connecting serial cables, serial protocol setups are required on the device side for some equipment such as the patient monitor (IntelliVue MP and MX series, Phillips North America Corporation, Andover, MA, USA), cardiac output monitors (FloTrac/Vigileo system and Vigilance monitor, Edwards Lifesciences, Irvine, CA, USA; CardioQ-ODM, Deltex Medical, Chichester, UK), bispectral index monitor (BIS Vista, Covidien, Dublin, Ireland), and target-controlled infusion pump (Orchestra^®^ Base Primea with module DPS, Fresenius Kabi AG, Bad Homburg, Germany).

**Table 2:** Supported devices and parameters.

Device	Type	Company	Parameters	Number of parameters	Data type	Acquisition interval (sec)
Solar 8000, Dash, MPS	Patient monitor	GE healthcare	Heart rate, blood pressures, oxygen saturation, temperature, gas concentrations, etc.	24	numeric	2
TramRac-4A	External module for patient monitor	GE healthcare	ECG, capnography, plethysmography, respiration, blood pressures	11	wave	dependent on the performance of analog-to-digital converter
IntelliVue MP and MX series	Patient monitor	Phillips	ECG, plethysmography, heart rate, blood pressures, oxygen saturation, temperature, gas concentrations, etc.	<100	wave and numeric	1/500 for ECG; 1/125 for pressure waves and EEG; 1 for numeric data
Primus, Fabius, Vamos, Zeus	Anaesthesia machine	Dräger	Gas concentrations, ventilatory volumes, flows, airway pressures	<90	wave and numeric	1/62.5 for waves, 7 for numeric data
Avance, Aestiva, Aespire	Anaesthesia machine	GE healthcare	Gas concentrations, ventilatory volumes, flows, airway pressures	<90	wave and numeric	1/25 for waves, 5 for numeric data
BIS Vista^TM^	EEG monitor	Covidien	EEG waves, bispectral index and related parameters	13	wave and numeric	1/128 for EEG wave, 1 for numeric data
Orchestra^®^	Target-controlled infusion pump	Fresenius Kabi	Target, plasma and effect-site concentrations; infused, residual, and total volumes; infusion rate and pressure; drug name and concentration	<10	numeric	1
Vigileo, EV 1000, Vigilance II	Cardiac output monitor	Edwards Lifesciences	Cardiac output and derived parameters, temperature, oxygen saturation,	<35	numeric	2
CardioQ-ODM+	Cardiac output monitor	Deltex	Stroke volume, cardiac output and related parameters	13	wave and numeric	1/180 for flow and arterial pressure waves; 1 for numeric data
INVOS^TM^	Cerebral/somatic oximetry	Covidien	Regional oxygen saturation	2	numeric	5
Belmont^®^ Rapid Infuser	Rapid infusion system	Belmont Instrument	Infused volume, infusion rate, temperature, pressure	7	numeric	every 2.875 mL infused

#### Data recording and editing

Once the connections between the medical devices and the serial ports of the computer running Vital Recorder is established, the Vital Recorder can start data recording. The recording duration is displayed at the top of the data-track window. Data recording intervals are device dependent, but the minimum acquisition interval for numerical data is typically 1 or 2 s. The time resolutions of the waveform data depend on the performance of the analog-to-digital converter, however 100 Hz for blood pressure or plethysmography and 500 Hz for electrocardiogram are frequencies generally obtained. The Vital Recorder writes data to the computer’s hard disk and it is recommended that the recorded data be backed up to the intranet-attached storage immediately. The stored data can be loaded and edited later in the Vital Recorder. Two or more fragmented files from one anaesthesia case can be seamlessly combined using the ‘merge’ function. The file size can be reduced by selecting and removing unnecessary data tracks from the track list. It is possible to erase unnecessary parts in the entire time length or to trim only the necessary parts. The data can be navigated rapidly using the mouse alone. Turning the mouse wheel on the track list scrolls the data tracks, and turning it in the track window zooms the data.

#### File format

The Vital Recorder saves the recorded data as a compressed binary file with ‘vital’ extension, which contains waves, numerical data, and event text. The standard GZip algorithm compresses one-hour data to less than 10 MB in size when seven 500 Hz waveform data, two 128 Hz electroencephalography (EEG) waveform data, and 90 tracks of numerical data are simultaneously recorded from the patient monitor, anaesthesia machine, BIS monitor, cardiac output monitor, and two infusion pumps. Gzip is a lossless compression algorithm that does not degrade the data quality. Further, there is no data loss due to floating-point operations when the waveform data is stored, because the count value passed from the analog-to-digital converter is stored as it is and the factors for physical unit conversions are stored separately.

The file type can be readily converted using the ‘export’ function that converts the original data format to various machine or human readable data formats such as csv, tsv, edf (European data format), HL7 (health layer 7), and mat (Matlab^®^ data format). The export function also has the options of adjusting the time intervals and handling the missing values. In addition, the Vital Recorder has an ability to import csv files with time and measurement data. The import/export functions of the Vital Recorder may facilitate data exchange with external systems such as statistical programs or other AIMS.

#### Program interface

**Figure 1:**
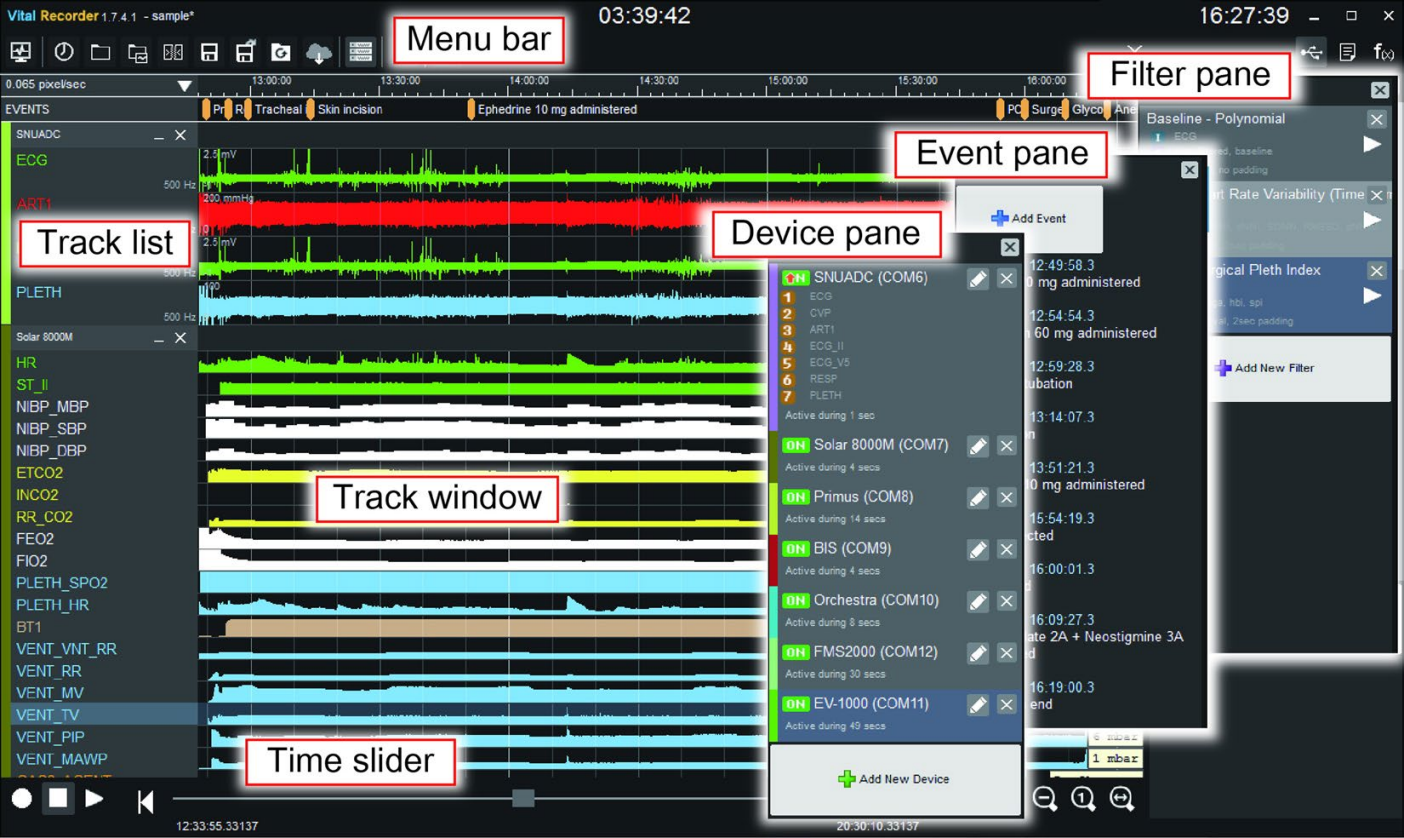
Schematic representation of the Vital Recorder. Track list shows all connected devices and their parameters. Input data are displayed in the track window in real time, and can be explored using the time slider and zoom buttons, or the mouse wheel. Three alternating panes are located on the right side of the data-track window in the track mode to support device setting, event management, and application of mathematical functions or medical algorithms.

The Vital Recorder has two switchable interfaces—the ‘track mode’ and the ‘monitor mode’. The device setting and data editing are performed in the track mode (Fig. 1). The track mode consists of the menu bar, track list, track window, and time slider on the left, and three alternating panes on the right. The data tracks show the input data from various medical devices, synchronised on the time axis. Zoom buttons and the time slider allow quick and convenient navigation through the entire data. The three panes are named device, event, and filter panes, and used for device setting, managing event markers, and applying various mathematical and statistical functions, respectively. Clicking the ‘Add New Device’ button in the device pane displays a categorised list of supported devices. After a device and the corresponding serial port are selected from the list, data tracks are automatically created and data is recorded. Registered devices are automatically reloaded when the program restarts. The event pane is especially useful for prospective research. An event can be added by clicking the ‘Add Event’ button, clicking on a time point in the event track, or simply pressing the Enter key. The details of the filter pane are described in *Advanced features*-*Filters* section.

**Figure 2:**
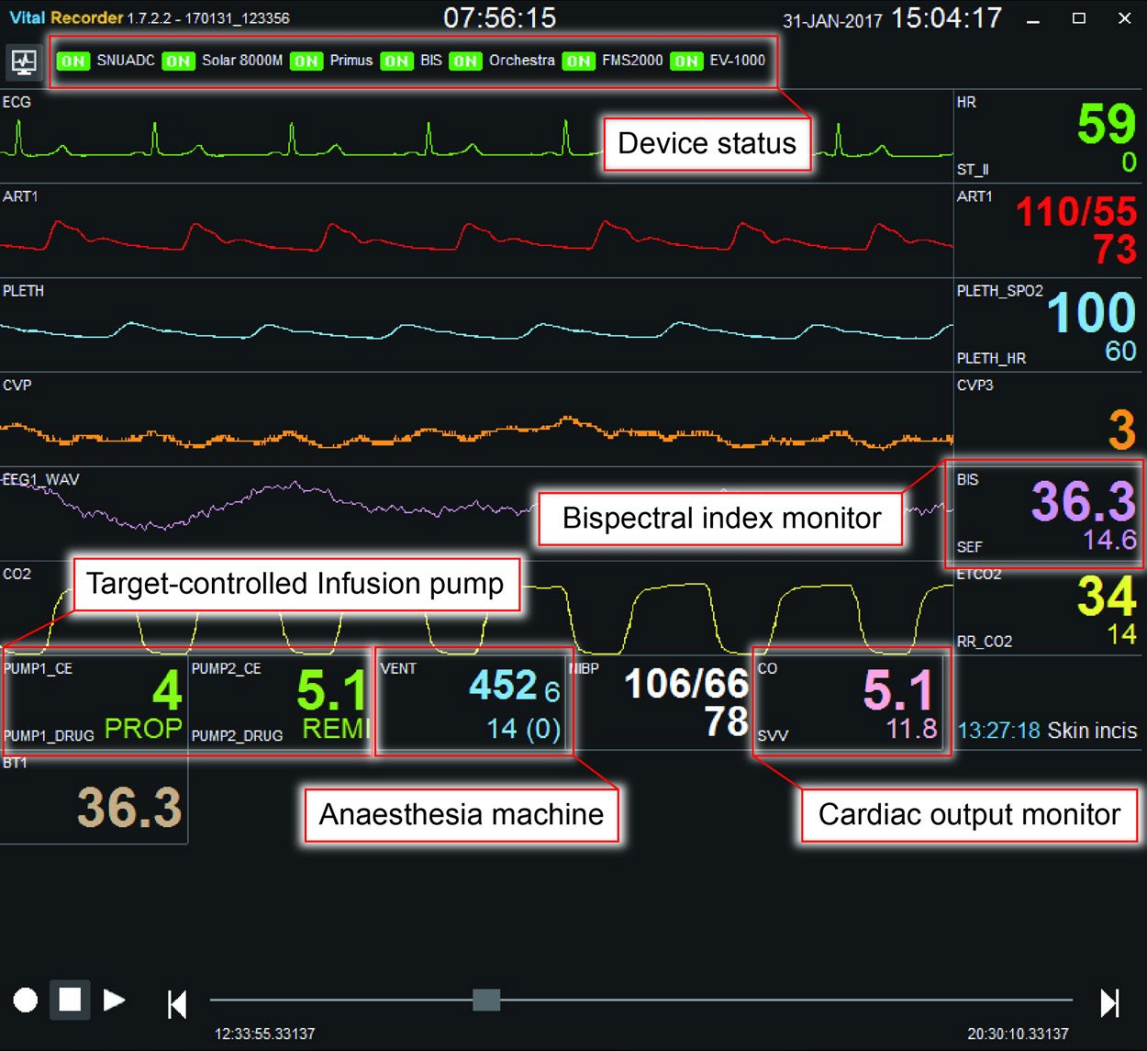
Monitor view of the Vital Recorder. The track and monitor modes are toggled by clicking the ‘monitor view’ button in the menu bar or pressing ‘CTRL + m’. The monitor mode is designed to monitor the status of data recording. The monitor mode shows the device connection status at the top, and simultaneously displays key data from multiple devices on a display that looks like a patient monitor screen.

The monitor mode is activated by clicking the ‘monitor view’ button in the menu bar, which displays a screen similar to the patient monitor screen (Fig. 2). The monitor mode is designed to monitor the status of data collection from multiple devices through the familiar patient monitor interface.

#### Advanced features

The Vital Recorder has some unique features that are not sufficiently supported in the previously reported data-collection programs or the commercial AIMS.

## Automatic recording

The connection between the program and medical devices may be temporarily cut off during data recording due to electrical noise or physical disconnection of the cable in the operating room. The Vital Recorder constantly checks the status of the communication ports and repeatedly attempts to recover the connection if data flow is interrupted. The automatic recording starts a new case if the heart rate and oxygen saturation signals are input more than five times within one minute, and stops 10 min after both signals disappear. There are options for automatically deleting invalid signals between cases and separating cases by date. These options support automated data collection with minimal effort, and help researchers build big data registries.

## Remote monitoring

The primary purpose of remote monitoring is to constantly monitor the status of data collection. Registered researchers can view the screen of the monitor mode of the Vital Recorder through a web browser. To enable remote monitoring, a unique room code issued by the Vital Recorder must be registered on the homepage, https://vitaldb.net. The vital signs data are then transmitted to the web server via the https protocol and stored for eight hours; the data can be reviewed retrospectively. However, the use of web monitoring for diagnostic purposes may be restricted by the law in some areas; therefore, this feature is recommended for research and educational purposes only.

## Filters

The filter function is designed to help researchers use the advanced computational functions or to test new algorithms easily. Filters written in Python language are installed in the ‘filters’ subdirectory of the Vital Recorder program. The filter function can be easily activated by clicking the ‘Add New Filter’ button in the ‘Filter’ pane and selecting the appropriate function. Filters work by defining input and output data tracks. For example, the Surgical Pleth Index (SPI) is calculated by assigning the plethysmography wave track as the input source and the ‘spi’ as the output data^17^. When the filter is run, a new ‘spi’ track with SPI data appears in the track window. Filters based on various mathematical, statistical, and medical algorithms are available and are constantly being added^18–21^. Researchers can also add their own filters to the ‘filters-user’ directory.

## File utilities

Four utility programs are provided to easily identify and extract the required data tracks from within many recorded files. After confirming the existence of key data tracks using the ‘vital_list.exe’, or checking the entire data tracks within the vital files using the ‘vital_trks.exe’, the required data tracks can be extracted from all cases at once using ‘vital_recs.exe’. If necessary, the ‘vital_deid.exe’ changes the record start time to January 1, 2100 for de-identification. These command line utilities allow researchers to create scripts that can batch-process many recorded files.

## Data bank

The collected vital signs data can be provided to users to support research, education, training, and engineering. By clicking the ‘Import from web database’ button in the menu bar, the Physionet database (http://physionet.org, accessed 14 February 2017), a public database of time-series physiological signals, can be accessed directly^22^. As the Vital Recorder supports a standard file format designed for exchange and storage of medical time-series data such as an edf file, the Physionet data can be downloaded, read, and analysed using the Vital Recorder. In addition, we plan to release the entire vital signs data collected by ourselves using the Vital Recorder to researchers who have difficulty in collecting physiological signals on their own. Currently, only a portion of this data is provided as sample files, after removing personal identifiers.

### Device connection and data collection

**Figure 3:**
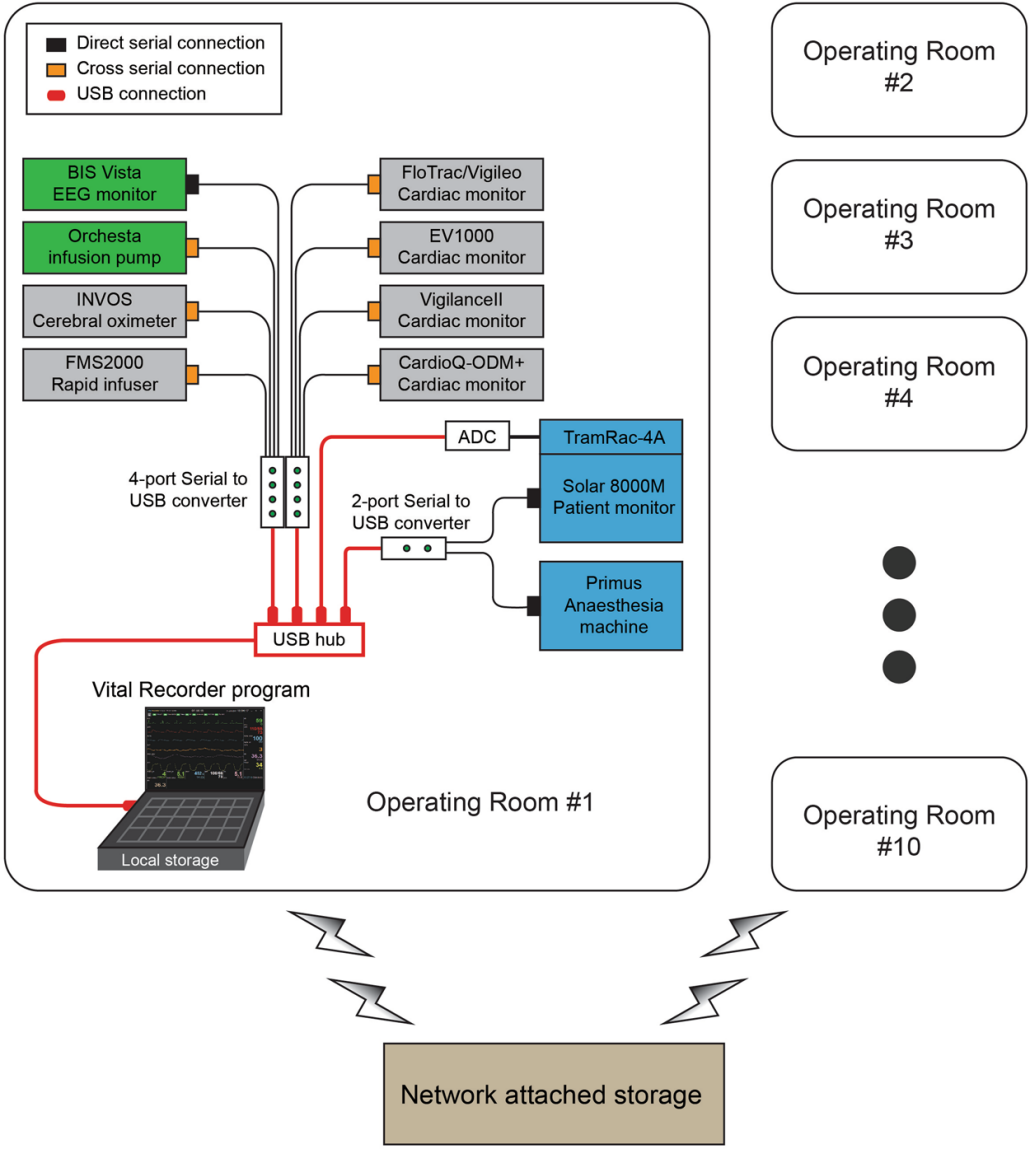
Schematic representation of device setup for data recording from multiple anaesthesia devices using the Vital Recorder. Data from a patient monitor, anaesthesia machine, and bispectral index monitor were simultaneously recorded. Target-controlled infusion pumps, cardiac output monitors, regional oximeter, and rapid infusion device were added as needed. Two or three serial-to-USB converters and one analog-to-digital converter (ADC) were used for data communication-port connections. A network-attached storage was used to back up the files from 10 operation rooms.

Intraoperative vital signs were recorded using the Vital Recorder, from June 2016 to January 2017. Fig. 3 shows the device connections for recording data using the Vital Recorder in operating rooms. The basic equipment used in the operating room were a patient monitor, anaesthesia machine, and BIS monitor. Target-controlled infusion pumps, cardiac output monitors, regional oximeter, and rapid infusion device were added as needed. Two or three serial-to-USB converters and one analog-to-digital converter (SNUADC; an Arduino-based analog-to-digital converter designed by authors; 8-channel, ± 5 V measurement range, 10-bit measurement resolution) were connected to a laptop computer via a USB hub. The automatic recording option was used exclusively. The recording of data was checked frequently through web monitoring. The recorded file was automatically saved in a folder, whose name was the date on which the file was created. Files recorded on the laptop’s hard disk were backed up to the network-attached storage using an automatic synchronization method provided by the storage vendor. The entire data were reviewed weekly, and the fragmented files and invalid files were merged and deleted, respectively.
